# TOO SICK TO BE TRUE? EVALUATING PROBLEMATIC CODING PRACTICES IN MEDICARE’S PATIENT DRIVEN PAYMENT MODEL

**DOI:** 10.1093/geroni/igae098.3907

**Published:** 2024-12-31

**Authors:** Harsha Amaravadi, Rachel Prusynski, Paul Fishman, Tracy Mroz

**Affiliations:** University of Washington, Seattle, Washington, United States; University of Washington, Seattle, Washington, United States; University of Washington, University of Washington, Washington, United States; University of Washington, Seattle, Washington, United States

## Abstract

The 2019 Patient Driven Payment Model (PDPM) aimed to promote budget neutral, patient-centered care by adequately reimbursing skilled nursing facilities (SNFs) for patient clinical complexity. Though PDPM mitigated financial incentives to provide excessive care, it may have introduced new incentives to falsely inflate or “upcode” clinical complexity. We hypothesize that upcoding may occur for common, chronic conditions associated with high reimbursement under PDPM. High reimbursement encompasses both diagnosis codes and services for complex conditions (e.g., diabetic wound care or IV use for severe anemia). Example conditions include complicated diabetes, blood loss anemia, and obesity. We sought to quantify the degree of documentation changes for conditions likely to be upcoded. Using a difference-in-difference design, we analyzed Traditional Medicare claims from 2018-2021 for all patients with a hospital-SNF care episode (n = 5.43 million), comparing diagnoses on the index hospital claim to the corresponding SNF claim for each patient. By using hospital claims, which are not subject to PDPM, as a control, we mitigate all patient-level confounding. We detected increased documentation for the selected conditions, representing an additional 32.18% [95%CI: 31.55, 32.82] in SNF coding for complicated diabetes, 54.59% [95%CI: 52.33, 56.95] for severe anemia, and 83.08% [95%CI: 82.00, 84.15] for obesity. PDPM sought to improve payment adequacy for caring for clinically complex patients, but Medicare’s financial sustainability also depends on accurate documentation of clinical factors that influence resource utilization and care provision. Our investigation of potential upcoding may inform future policy refinement to support sustainable, patient-centered care delivery for older adults.

